# Reviews of Books

**Published:** 1923-12

**Authors:** 


### REVIEWS OF BOOKS.

### PSYCHOLOGICAL MEDICINE.

**A Manual of Psychotherapy.** By Henry Yellowlees, O.B.E., M.D. (A. and C. Black. 10s. 6d. net.)

The author of this book, which we may say at once supplies a real need, bears a name which is honoured in the history of psychological medicine, and has himself great experience in that speciality. The student of medicine has had, for years past, to attend lectures and demonstrations on insanity. But there is a growing demand that the present requirements should be extended, and that the curriculum should include a course of instruction upon psychotherapy in general. A knowledge of this subject would be far more useful to the general practitioner than the details of many departments of medical practice which the student is compelled laboriously to acquire. The necessity for the possession of this knowledge will increase, for psychotherapy, if no longer in its infancy, is still in its childhood. Dr. Yellowlees has produced an excellent summary of the elementary aspects of the subject which will assist the practitioner to use psychotherapy in the many cases occurring in ordinary practice which call for this form of treatment, and will help him to recognise the cases which require specialist help.

At the outset, Dr. Yellowlees urges the reader to think about these matters in psychological terms, warning him that nothing but confusion can, at present, result from mixing psychology and physiology. He gives a brief summary of the theory of the unconscious mind, and of the nature of repression, a subject which is of supreme importance in the study of psychotherapy. An admirable account of the mental mechanism of sublimation, fantasy, rationalization and other processes follows, and after this the author takes up the question of instinct and conflict. He is most careful to explain the very extended sense in which the term " sex " is used by the Freudian school, this being a point upon which there is much misapprehension. Symbols and dreams are discussed in an elementary but lucid fashion.

The second part of the book is concerned with the various methods used in psychotherapy. The difference between the processes of suggestion and persuasion is clearly pointed out, and there is an illuminating discussion of the theory of suggestion. An account of hypnotism is given in the course of which the author refers to the violent opposition aroused by hypnotism in its inception. The quotations which the author gives, amazingly absurd as they appear to us, can easily be paralleled in the outpourings of certain present-day writers who, while never having concerned themselves to learn anything about the matter, have violently attacked the newer psychological theories. There is a short but adequate account of psycho-analysis, regarded both as a therapeutic method and as a system of psychology, for, as is pointed out, the two aspects are distinct. We may hope that this short introduction will induce some readers to pursue the study into larger works on the subject. Dr. Yellowlees appears to be an orthodox Freudian, but on dream interpretation he writes most fairly, and gives due weight to the other theories of dreams.

Finally, the scope of psychotherapy is considered, and a good classification of " nervous diseases " is given. The author then takes up the application of psychotherapy in the cure of neurasthenia, hysteria and anxiety states. Useful rules are given for the employment of psychotherapy in general practice, with excellent illustrative cases. We doubt, however, whether psycho-analysis will ever be possible in general practice, although other methods of psychotherapy are, no doubt, feasible. But it is well that the student and practitioner should learn what can be done, in this direction, by competent specialists. The whole book is admirably written. No better preliminary survey has yet appeared, and we congratulate Dr. Yellowlees upon the skill with which he has prepared so lucid an introduction to a most difficult and complicated subject.

### FOOD AND HEALTH.

**Vitamins: A Critical Survey of the Theory of Accessory Food Factors.** By Ragnar Berg, Director of Physiological Research, Weisser Hirsch. Translated by Eden and Cedar Paul. (Allen and Unwin. 18s. net.)

Professor Berg has written a valuable survey of the deficiency diseases and the accessory food factors, the deficiency of which is their cause. This critical survey is all the more valuable because it seems apparent that Professor Berg reluctantly accepts the proof that our food contains other items of importance than the proteins, fats, carbohydrates and inorganic salts. He is a physiological chemist of repute, not a practising physician, but his acumen and physiological training bring him into the first rank of observers. Though we may be unable to assimilate the whole of his views we are bound to accept them in part, and even a partial acceptance implies that the absence of vitamins is not the only factor in the causation of the deficiency diseases. A physiological chemist of such rank would naturally first examine the food of the sufferer for deficiencies of the known food factors before searching for the unknown; and such close examination of the food as he has given throws a strong light on the causation of growth, scurvy, rickets, osteomalacia, osteoporosis, psilosis or sprue, pellagra, the œdema of malnutrition, beriberi, and other forms of polyneuritis.

The conditions of growth are defined by him. Children and young animals must be given a sufficiency of food; it must contain a due proportion of protein of a high value, and from the point of view of protein, milk and potatoes are the best. Cereals are less suitable except when eaten with milk. The food must contain enough fat-soluble A, which can most advantageously be supplied in milk, butter, spinach and tomatoes; and water-soluble B and C, which are found in fresh fruit and fresh vegetables. Care must be taken that the salts of sodium, potassium, calcium and magnesium are present in due proportion. With regard to protein, he finds, as others have found, that lysin and tryptophan are amino-acids essential for growth and maintenance, and these are not found in vitamins, and therefore vitamins do not supply that deficiency. When these amino-acids are absent from any article of food, such food is incomplete and a cause of one or other of the deficiency diseases. Eggs contain an abundance of lysin and tryptophan; milk protein is also adequate for growth and of extraordinarily high nutritive value; meat is the best source of protein for human beings and is adequate for growth and maintenance. But maize is deficient in the lysin and tryptophan so essential for life; rye also lacks lysin, is deficient in tryptophan, and is inferior to barley. Rice likewise is deficient in growth-producing elements. Wheat has two proteins—glutenin, which is adequate for growth, and gliadin, which is deficient in lysin, and therefore is not competent sufficiently to promote growth or maintain weight. Therefore bread, especially white bread, is defective unless it is accompanied by the higher grade proteins of meat, eggs, milk or nuts.

But the diet for normal health must contain a sufficiency of all necessary ingredients, and there must not be a notable excess of any. It is the very keenness of Professor Berg's insistence on the importance of the proper quality of protein, the due proportion of fat and carbohydrate, and the presence and correct mixture of the essential inorganic constituents in our food that makes this book valuable. It is a mine of information concerning all these points, and if he lays stress upon the importance of proteins and salts, he admits the essential nature of the vitamins or complettins, the characters, composition, source and effects of which he so admirably depicts. A word of appreciation should be accorded the translators, who have done their work well. The bibliography is extensive and of high value.

### WHERE MEDICAL EXAMINATIONS BEGAN.

**The School of Salerno** (Magistri Salernitani Nondum Cogniti). Wellcome Historical Medical Museum Studies, No. 2. By Dr. Pietro Capparoni. John Bale, Sons, and Danielsson. 15s. net.

This volume, which is the second in the series of Research Studies in the History of Medicine, published under the auspices of the Wellcome Historical Museum, owes its origin to the chances of war. Dr. Capparoni, the General Secretary of the Italian Medical History Society, found himself stationed during the war at the town of Salerno, some thirty miles south of Naples. Taking advantage of his opportunity, he made a careful investigation in the archives of the Cathedral of St. Matthew, with special reference to the great school of medicine which formerly existed there.

Though its origin is lost in antiquity, throughout the Middle Ages the Medical School of Salerno enjoyed a reputation higher than that of any other seat of learning. Kings and princes, bishops and nobles, travelled from far-distant places to seek the advice and aid of the greatest medical teachers of their time. William of Normandy, afterwards to be the victor of Hastings, spent some time there to be cured of wounds, as also did his son, Robert of Normandy. In 984 A.D. Alberon, Bishop of Verdun, and the Abbot Desiderius, afterwards Pope Victor III., both sought restoration to health amid its bracing air and lovely scenery. There appears to be little doubt that at Salerno the medical lore of Greece and Rome found a meeting-place with the vivifying mind of the Arabian physician. Moreover, lying on the great route traversed by the Crusaders, Salerno served as a kind of Base Hospital for the war-worn knights who had suffered for the Cross in the Holy Land.

The great reputation which the School achieved naturally brought students from countries as far distant as Scotland, England and Germany, and gave force to the description written by Petrarch, *Medicinæ Fons ac Gymnasium nobilissimum.* Thus in the volume of MSS. specially examined by Dr. Capparoni are to be found the names of sixty-three Normans and English who had gone thither for study. Such a concourse of students and teachers naturally led to the foundation of a Gild or Confraternity for the better regulation of the University, as well as for the mutual help and well-being of its members. It was here that medical examinations as necessary antecedents to practice were first instituted, while the teachers enjoyed a freedom from the necessity of paying taxes—a privilege which might well be reintroduced in these hard times.

Like the Gild of Barber-Surgeons in England, this confraternity was partly religious in character, and it is to this fact that the two MSS. studied by Dr. Capparoni owe their origin. One is the *Liber Confratrum,* or list of members of the Confraternity of the *Cruciati;* the other an obituary list of those members whose memory it was desired to perpetuate. Some seventy years ago de Renzi published a list of 13,000 names associated with the School. To these Dr. Capparoni has been able to add forty-five previously unknown. But of greater interest to us are the biographical details which his wide historical knowledge has enabled him to collect concerning many of them.

As other rival Universities arose, Salerno gradually sank into insignificance, until the *coup de grâce* was given by Napoleon, who suppressed it in 1811. Perhaps the School had survived its usefulness, but an institution which had existed for a millennium deserved a better fate. The book is illustrated by seventeen finely reproduced photographs, chiefly of the MSS. which are preserved in the Cathedral of St. Matthew. Sir D'Arcy Power supplies a short and interesting Preface.

### WHERE TO LIVE FOR HEALTH.

**Medical Climatology of England and Wales.** By Edgar Hawkins, M.A.Oxon., M.D.Edin., D.P.H. (H. K. Lewis & Co. 25s. net.)

The question, "Where shall I send my patient to live?" has constantly to be answered by the medical man, who is expected to be able to name off-hand at least half-a-dozen or so places suitable for this or that disease or tendency thereto. The much-abused formula, "Change of air," is very easy to pronounce, but it requires some little knowledge of climatology and physical geography to be able to prescribe residence in a given locality with as much precision as one would order mercury or sulphur.

In the present volume Dr. Hawkins has appreciated the difficulties of the student and practitioner, and has succeeded conspicuously in presenting the main facts concerning the climate of all the principal resorts in England and Wales in a clear and comprehensive fashion. The Blue Books issued by the Meteorological Office have been largely drawn upon by the author, together with the *Monthly Summaries* and other official documents. To render the principal climatological factors of a given resort clearly, a system of graphic records has been adopted throughout the book, the majority of these being worked upon the comparative percentage basis. The eye is, therefore, enabled to take in at once the main climatic features of, say, Margate or Torquay, by reference to such a diagram. The countries with which this book deals are divided into seven districts, of which there are many coloured synoptical charts. The mean monthly variations of each district, as well as of the principal resorts in that area, may also be studied in graphic form, first as regards their actual recorded values, and next as showing their values as percentages of the normals.

A valuable section of the book deals with the therapeutics of the English climate, of which two main classes are described—the tonic or bracing, and the sedative or relaxing. These terms should, of course, be regarded as relative to the place in which the patient is already residing. The appropriate climates are here given for rheumatism, anæmia, scrofula, nervous disorders, etc. The index itself is so arranged that it constitutes a therapeutic guide, and there will be found the unique feature of a "warning" against sending patients affected with certain maladies to those places thus indicated. While it is to be regretted that, owing to limitations of space, and also upon economical grounds, it has not been possible to include Scotland and Ireland, we have no hesitation in saying that, for England and Wales, Dr. Hawkins's book is the most suitable and trustworthy climatic guide we have come across.

### MISCELLANEOUS NOTICES.

### Anæsthetics.

Handbook of Anæsthetics. By J. Stuart Ross, M.B., Ch.B., F.R.C.S.C. (Livingstone, Edinburgh. 12s. 6d. net.)—A second edition of a useful volume, the result of much practical experience. A certain amount of new material has been added, and the whole book has been revised and brought up to date.

### The Registered Nurses of Scotland.

The Register of Nurses Kept by the General Nursing Council for Scotland. (The G.N.C. for Scotland. 5s.)—Well bound in blue and clearly printed on good paper, the Register contains the names, qualifications and addresses of all nurses registered at December 31, 1922, under the Nurses Registration (Scotland) Act of 1919.

### The Nurse in the X-Ray Department.

Notes on Radiology for Nurses. By Harold Wigg. (The Scientific Press. 1s. 3d. net.)—It is the author's aim to provide the hospital nurse with the initial instruction necessary to enable her to carry out her duties in the X-Ray department and to avoid the pitfalls which beset the beginner. Simply written and excellently illustrated, every nurse would do well to possess this little book.

### Making Marionettes.

Marionettes and How to Make Them. By F. J. McIsaac. (Stanley Paul. 2s. 6d. net.)—The making of marionettes should be a fascinating occupation to any child with capable fingers, and this little book, with its clear directions and numerous illustrations by Mr. Tony Sarg, should be a welcome Christmas present. Any owner of this book is entitled to enter a competition in which 500 prizes are to be given away for the best pictures, plain or coloured, of a marionette theatre.

### A New Book of Medical Reference.

London Doctors and Dental Surgeons. (Grafton Publishing Co. 15s. 6d.)—The first issue of a handy book of reference containing the names of all doctors and dental surgeons practising in Greater London, giving, in addition to qualifications, etc., such useful practical details as telephone numbers and consulting hours. The volume ranges in shape with the Medical Register, and the General Medical Council has officially notified the publishers that it conforms to its requirements. It promises to be a very valuable reference volume.

### Dental Mechanics.

Golden Rules of Dental Mechanics. By Harold Osborn. Second Edition. John Wright and Sons, Ltd., Bristol. 5s. net.—This is a particularly handy little book for those interested. It sets out to give only useful hints, notes and data, instead of a complete survey of the subject, and this purpose it achieves uncommonly well. There is no phase of dental mechanics upon which some wise saying is not included, and the book is not, as are so many of these summaries, difficult and tiresome to read.

### How to Feed the Sick.

The Art of Feeding the Invalid and Convalescent. By a Medical Practitioner and a Lady Professor of Cookery. Revised and brought up to date by Jessie J. Williams, M.C.A. (The Scientific Press. 4s. net.)—The importance of an attractive and nourishing diet for invalids cannot be over estimated, but it is open to serious doubt whether the average hospital menu is yet all that it should be. It is often much lacking in variety and is distinctly uninspired, while too often the Sister in charge of the department has no particular qualification for her post. To find tempting dishes for a convalescent child or for a chronic dyspeptic is an unenviable task, and any help which can be given in this direction is likely to be welcomed. Miss Williams has brought this publication thoroughly up to date and in harmony with modern dietetic standards; and it contains numbers of excellent recipes as well as suggestions for food in indigestion, gout, diabetes and consumption. As a guide to "invalid country" it could not be bettered.

### A Hospital Quarterly.

Guy's Hospital Reports. No. 4. October, 1923. (Henry Frowde and Hodder and Stoughton. Quarterly. 12s. 6d.)—The October number of these reports opens with a carefully compiled description of the life and work of the late Sir George H. Savage, who first entered Guy's in 1861. This is followed by two papers discussing the relation between tonsillar infections and rheumatism. In the first Dr. G. H. Hunt, Physician to the Hospital, describes the effects in a considerable number of children who had been in the hospital with acute rheumatism. In some of these the tonsils had been enucleated in the hope that recurrence might be prevented. In others no such operation had been performed. From a careful analysis of the series Dr. Hunt concludes that he cannot support the contention that removal of the tonsils diminishes the liability to rheumatic infection, and that this operation on rheumatic children whose tonsils and adenoids are normal is quite unjustifiable. The second paper, by Dr. H. J. Starling, of Norwich, argues the opposite side of the question. A practical point is raised by a note of experiments by Dr. F. A. Knott in testing the effects of gastric juice upon those bacteria commonly swallowed. It is shown that free hydrochloric acid is the only essential lethal agent, and that its germicidal power upon the different strains corresponds closely to the frequency with which infection viâ the mouth is known to occur. The marked destructive effect of free HCl. upon pyogenic micrococci must account for the apparent immunity of certain people with obvious oral sepsis, and it appears that before accounting for all a patient's troubles by condemning his teeth, tonsils, etc., it would be well to know the result of a test meal and his customary strength in gastric hydrochloric acid.

